# Genetic loci regulating arsenic content in rice grains when grown flooded or under alternative wetting and drying irrigation

**DOI:** 10.1186/s12284-019-0307-9

**Published:** 2019-07-22

**Authors:** Gareth J. Norton, Anthony J. Travis, Partha Talukdar, Mahmud Hossain, Md Rafiqul Islam, Alex Douglas, Adam H. Price

**Affiliations:** 1 School of Biological Sciences, University of Aberdeen, Aberdeen, AB24 3UU Scotland; 2 Department of Soil Science, Bangladesh Agricultural University, Mymensingh, Bangladesh

**Keywords:** Rice, Arsenic, Genome wide association genetics, Alternate wetting and drying

## Abstract

**Background:**

Rice is a global staple crop, being the main calorific component of many people living subsistence livelihoods. Rice can accumulate toxic elements such as arsenic, with the crop water management strongly affecting uptake. This study utilises the Bengal and Assam Aus Panel to conduct genome wide association (GWA) mapping for arsenic in shoots and grains of rice grown over 2 years under continually flooded (CF) and alternate wetting and drying (AWD). The aim was to assess genotype by water management interaction, identify quantitative trait loci (QTL) for arsenic accumulation, and propose candidate genes for lowering grain arsenic.

**Results:**

AWD significantly reduced grain arsenic across all cultivars on average by 15.7 and 15.1% in year 1 and 2 respectively and shoot arsenic by 27.0% compared to the plants grown under CF. There was a weak cultivar by treatment interaction for grain for arsenic.

All traits were strongly influenced by cultivar. GWA mapping identified a large number of 74 individual QTLs for arsenic, with six QTLs showing stability across years and/or water treatments. Three of the loci (one on chromosome 3, one on chromosome 4, and one on chromosome 5) were investigated in detail using an approach of clustering cultivars that had similar haplotypes for the QTL regions and then looking at the phenotypic values across the clusters. Two of the identified QTLs co-localised with known genes involved in arsenic accumulation, including *Lsi2* which has not previously been reported to underlie a grain arsenic QTL.

**Conclusions:**

This study has identified a number of novel QTLs for arsenic accumulation, as well as cultivars that consistently accumulate less arsenic over multiple field traits. The use of a haplotype clustering approach after GWA mapping has allowed for the effect, in terms of arsenic accumulation, to be determined for cultivars that share similar genomic sequence. Allocating nine high yielding Bangladeshi cultivars to these clusters has identified the potential of utilising these QTLs in breeding programmes; for example, incorporation of the QTL on chromosome 5 should decrease grain arsenic in elite high yielding Bangladeshi cultivars by 10% in all high yielding cultivars studied.

**Electronic supplementary material:**

The online version of this article (10.1186/s12284-019-0307-9) contains supplementary material, which is available to authorized users.

## Background

The main challenge in rice (*Oryza sativa* L.) production in the future will be increasing production without increasing land use, using inputs (e.g. fertiliser, water) more efficiently, and coping with climate change in the form of increased biotic and abiotic stresses. While addressing these issues of quantity of production, it has become increasingly apparent that attention needs to be given to the quality of rice produced, where excess in toxic elements such as arsenic can cause concern.

Arsenic is a class one carcinogen and several studies have highlighted the issues of arsenic content in rice (e.g. Mondal and Polya 2008; Meharg et al. 2009; Zhao et al. 2010a). In 2016 the European Union (EU) regulated inorganic arsenic content in rice, but 1 year after the regulation was introduced, nearly all rice-based products specifically marketed for infants and young children had arsenic concentrations over the limit (Signes-Pastor et al. 2017).

Arsenic in soils can be present due to natural geogenic factors and can also be influenced by a wide range of anthropogenic activities. Of particular concern is the ability of rice to accumulate higher concentrations of arsenic compared to other staple cereals (Williams et al. 2007). This elevated accumulation is primarily due to the dominant practise of flooded paddy cultivation (Xu et al. 2008; Norton et al. 2013). Under flooded conditions inorganic arsenic is predominantly present as arsenite whereas in aerobic soils arsenic is present as arsenate (Dixit and Hering 2003). Arsenite has a greater mobility compared to arsenate and they are accumulated within the plant through different transport systems. Arsenate is transported through phosphate transporters (Meharg and Hartley-Whitaker 2002), while arsenite is transported though silicon transporters (Ma et al. 2008; Zhao et al. 2010b).

Previous studies have proposed a range of mechanisms for decreasing the accumulation of arsenic (Brammer 2009; Chen et al. 2017). One such approach is the management of water whilst growing rice (Chou et al. 2016; Linquist et al. 2015; Somenahally et al. 2011; Norton et al. 2011; Norton et al. 2017a; Norton et al. 2017b; Xu et al. 2008; Norton et al. 2013). Cultivating rice under flooded conditions affects the soil chemistry, which in turn affects the availability and uptake of many elements (Rinklebe et al. 2016). It has been observed that grain arsenic is approximately 10 times lower in rice grains when plants were grown under non-flooded conditions compared with flooded conditions (Xu et al. 2008; Norton et al. 2013; Norton et al. 2011; Zhang et al. 2014). However, aerobic rice production cannot currently meet the sustained yields of flooded rice (Belder et al. 2005; Atwill et al. 2018). Under alternate wetting and drying (AWD) water management (a technique where the soil cycles through flooded and non-flooded periods) grain arsenic was reduced by 14–26% (Norton et al. 2017a).

Another strategy for producing rice grain with less arsenic is breeding; studies of multiple cultivars have repeatedly demonstrated variation in the range of 3–34 fold for flooded rice (Norton et al. 2009a; Norton et al. 2009b; Norton et al. 2011). Genetic mapping studies have identified quantitative trait loci (QTLs) that are responsible for arsenic content (Norton et al. 2014; Norton et al. 2012; Norton et al. 2010; Zhang et al. 2014). However, in many cases the confidence intervals of the QTLs are large and identification of the gene(s) responsible for the phenotypic variation is not feasible. Additionally, identification of candidate genes can be difficult as different loci are revealed when the same population is tested in different sites or indeed in the same site over different years (e.g. Norton et al. 2014). Another issue complicating genetic mapping for grain arsenic is the fact that flowering time influences the trait (Norton et al. 2012; Norton et al. 2011), probably because of time-dependent variation on arsenic availability in soils. The reasons described above contribute to the challenge of identifying reliable loci that breeders will have confidence in.

Gene mutation, knockout, and overexpression studies have demonstrated variation in the accumulation of arsenic in rice grains (e.g. Ma et al. 2008 and Song et al. 2014). However, these can produce other undesired effects. For example, the arsenite transporter, Lsi2, was characterised in a knockout mutant that had substantially reduced growth and yield (Ma et al. 2007). To date, these genes have not been reported to underlie grain arsenic QTLs either because there is not sufficient allelic variation for them within rice, or because the mapping populations that have been used cannot reveal it.

Here, the genetically and geographically focused rice diversity resource the Bengal and Assam Aus Panel (BAAP) was deliberately selected to have a narrow window of flowering so grain-related traits could be better studied (Norton et al. 2018). Plants were grown under both continually flooded (CF) conditions and under the water saving technique of AWD in a field experiment in Bangladesh over two seasons. The population has been genotyped with approximately 2 million SNPs, which allows for association mapping to be conducted for any measured trait (Norton et al. 2018). The aim of the study was to assess the impact of AWD on the accumulation of arsenic in the rice shoots and grains, to accurately identify genetic loci associated with arsenic accumulation, identify candidate genes within those loci, and determine if QTLs might be useful in breeding.

## Results

### Arsenic content

**Fig. 1:**
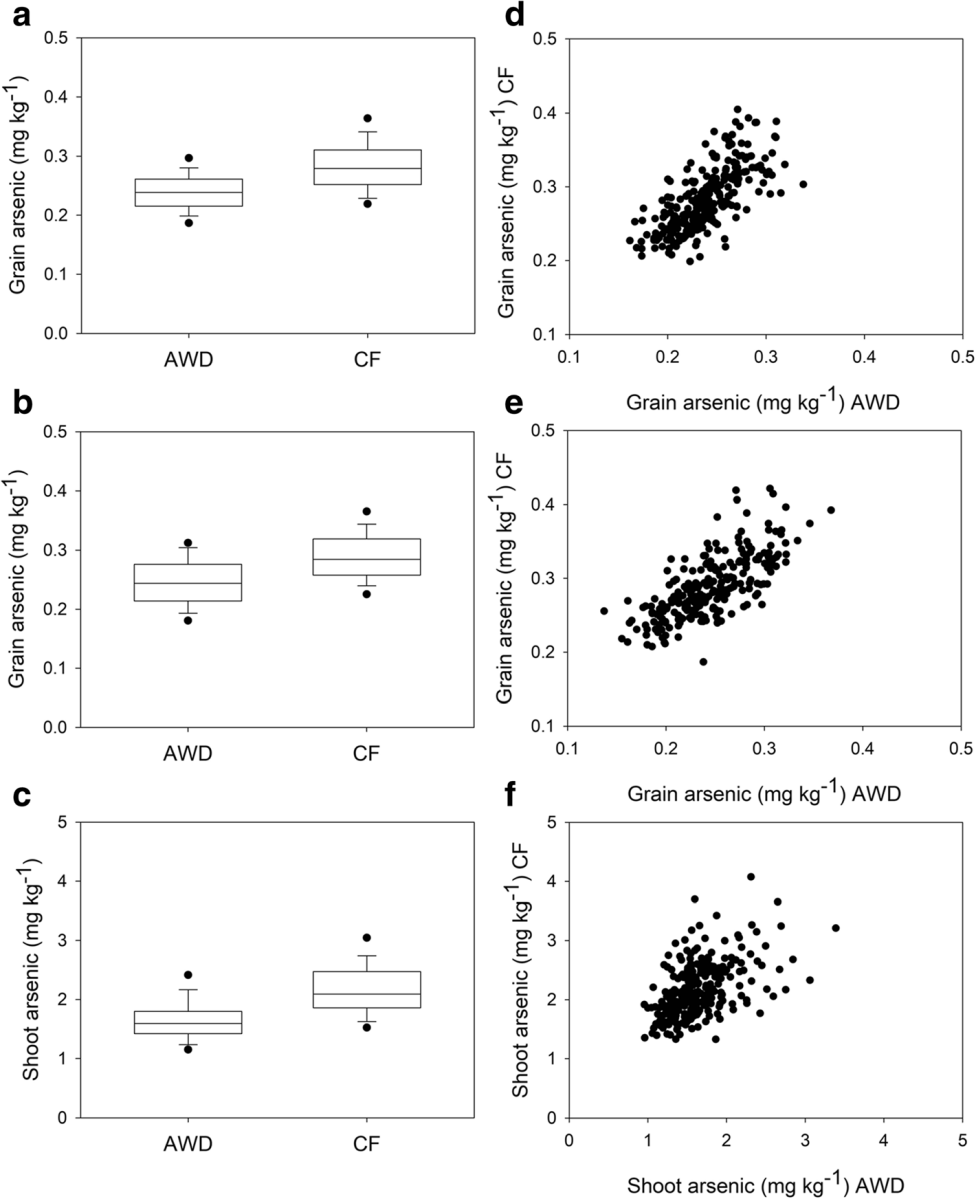
Grain arsenic 2013 (**a**), 2014 (**b**), and shoot arsenic (**c**). Correlations grain arsenic 2013 (**d**), 2014 (**e**), and shoot arsenic (**f**)

Grain and shoot arsenic concentrations data are presented in Table 1 while the mean values for each genotype is given in Additional file 1: Table S1. All traits were significantly different between the plants grown under AWD and CF in both years (Table 2). AWD reduced grain arsenic across all cultivars on average, by 15.7 and 15.1% in year 1 and 2 respectively, and shoot arsenic by 27.0% (Fig. 1, Table 1). Across all experiments there were genotypic differences for grain and shoot arsenic (Table 2), with genotypic differences explaining 42.4% of the variation observed in the grain phenotype in both years and 31.1% of the variation in shoot arsenic. In year 1, genotype by treatment interactions were observed for both grain and shoot arsenic concentration. For both grain and shoot arsenic there were strong correlations for arsenic concentration when the material grown under AWD and CF in each year was compared (Fig. 1). Eleven cultivars were consistently found to have low grain arsenic (Table 3), with SORIBHOG and Jhona 349 being consistently in the lowest 10% for grain arsenic across all experiments.

**Table 1:** Descriptive statistics for the grain and shoot arsenic concentration measured in 2013 and 2014 for the accessions grown under AWD and CF

			Min	Max	Median	Mean	SD
Grain As (mg/kg)	2013	AWD	0.161	0.338	0.239	0.239	0.032
		CF	0.199	0.405	0.279	0.283	0.042
Shoot As (mg/kg)		AWD	0.957	3.390	1.655	1.593	0.383
		CF	1.326	5.458	2.090	2.182	0.499
Grain As (mg/kg)	2014	AWD	0.137	0.368	0.244	0.245	0.041
		CF	0.187	0.421	0.284	0.289	0.043

**Table 2:** Two-way ANOVA results (F-values) for treatment, genotype, and the treatment x genotype interaction for the grain and shoot arsenic concentrations across 2013 and 2014. Numbers in brackets are the percentage contribution for that factor to the overall variation observed

		2-way ANOVA		
Year	Trait	Treatment	Genotype	Treatment x genotype interaction
2013	Grain As	758 *** (17.9%)	7.07 *** (42.4%)	1.30 ** (7.7%)
	Shoot As	472*** (14.7%)	3.92 *** (31.1%)	1.28 ** (10.1%)
2014	Grain As	466 *** (13.7%)	6.44 *** (42.4%)	NS

* *P* < 0.05, ** *P* < 0.01, *** *P* < 0.001

**Table 3:** Cultivars with consistently low grain arsenic in three or four of the experiments. An X indicates that that cultivar was in the lowest 10% for grain arsenic concentration in that experiment

		Year 1		Year 2	
BAAP Id	Cultivar name	AWD	CF	AWD	CF
4	ARC 5977		X	X	X
87	AUS 440	X	X	X	
90	AUS 462	X	X		X
107	AUS PADDY (WHITE)		X	X	X
157	PADMA SAIL	X		X	X
164	SORIBHOG	X	X	X	X
205	Jhona 349	X	X	X	X
220	Ghorbhai	X	X	X	
225	Karkati 87	X		X	X
265	P 79	X		X	X
271	99216	X	X	X	

### Relationship between arsenic and flowering time

Flowering time data has already been reported for this experiment (Norton et al. 2018), where a large majority of cultivars fall into a discrete flowering time window. There is no impact of phenology on shoot arsenic or on grain arsenic under AWD in year 1. However, for grain arsenic under AWD in year 2 and under CF in both years there is (Additional file 4: Figure S1). In all cases there was a significant negative correlation between the flowering time phenotype and grain arsenic (CF year 1, *R*^2^ = 5.6%, *p* < 0.001; AWD year 2, *R*^2^ = 10.6%, *p* < 0.001; CF year 2, *R*^2^ = 10.3%, *p* < 0.001). The arsenic traits reported here were not related to yield or biomass traits previously reported for this experiment (Norton et al. 2018).

### Arsenic GWA mapping

**Fig. 2:**
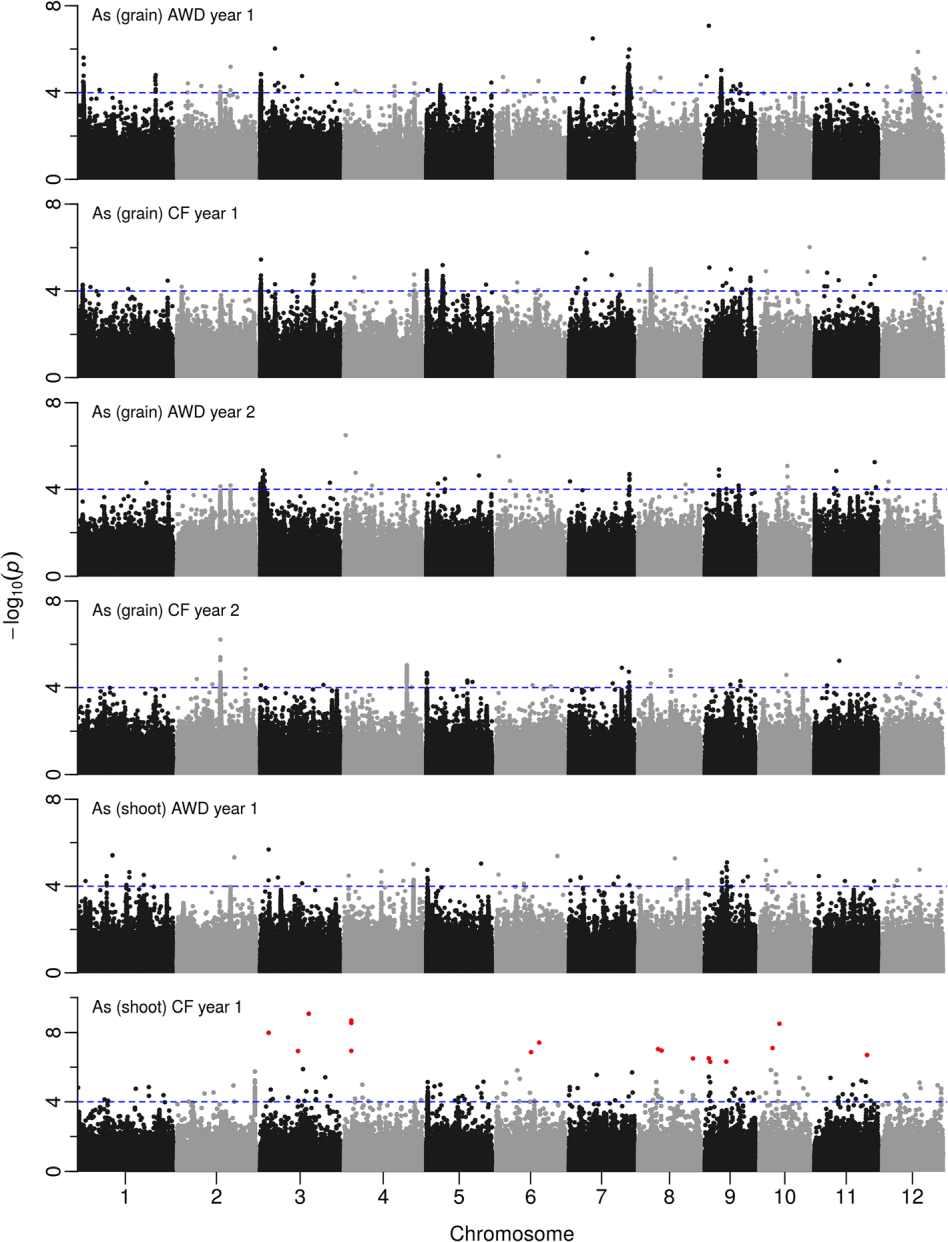
Manhattan plots from GWA mapping of shoot and grain arsenic in field experiments under AWD (Alternate Wetting and Drying) and CF (Continuous Flooding) over years 1 and 2. A guide-line in blue is shown at -log10(0.0001) = 4. Benjamini-Hochberg adjusted probabilities > 0.1 are highlighted in red

Genome wide association (GWA) mapping was conducted on the BAAP to identify genomic loci that control arsenic accumulation in rice plants. As nearly 1,500 significant SNPs were associated with the traits (Fig. 2), CLUMP was used to identify multiple SNPs that represent a single QTL. Note that singletons (SNPs associated with a trait, but with no other significant SNP within user-defined LD (linkage disequilibrium) decay) were not considered QTLs when using the CLUMP analysis. Once a QTL was identified, local LD decay was conducted to determine the region that the gene responsible for the QTL is likely to be within. Finally, an approach of genome clustering was applied for three QTLs that were investigated in detail. This approach led to the identification of clusters of genotypes that share a similar genome sequence within the QTL region.

Using GWAS followed by CLUMP, 74 QTLs were detected for grain and shoot arsenic content across the 2 years of the experiment, although 23 of these had only two significant SNPs (Additional file 2: Table S2). The number of QTLs detected for individual traits was as follows (with the number having greater than two significant SNPs in brackets); 21 (18) for AWD year 1 Grain, 10 (9) for CF year 1 Grain, 6 (4) for AWD year 2 Grain, 7 (4) for CF year 2 Grain, 15 (5) for AWD year 1 Shoot and 15 (11) for CF year 1 Shoot. These include QTLs where either several SNPs were detected in a single year, single trait, and single water condition; where the same loci was detected under different conditions, in multiple years; or for both shoot and grain content. Grouping individual trait QTLs based on being within the global LD decay value of 243 kbp, six loci were revealed where QTLs were detected in multiple treatments or years, being at 19.7 Mbp on chromosome 2, around 0.5 Mbp on chromosome 3, 0.4 and 6.4 Mbp on chromosome 5, 27.5 on chromosome 7, and 16.1 on chromosome 9 (Additional file 2: Table S2). Three of these loci were selected for additional detailed analysis. The region on chromosome 3 at ~ 0.34 Mbp was selected as the QTL region encompasses *Lsi2*, a known arsenic transporter (Ma et al. 2008). The the QTL on chromosome 5 at ~ 0.32 Mbp.was selected as it appears to be a grain specific QTL. A third loci was selected on chromosome 4 at ~ 31.7 Mbp as this loci which is co-localised with a gene involved in arsenic transport ABCC1 (Song et al. 2014).

### Chromosome 3 ~ 0.34 Mbp

**Fig. 3:**
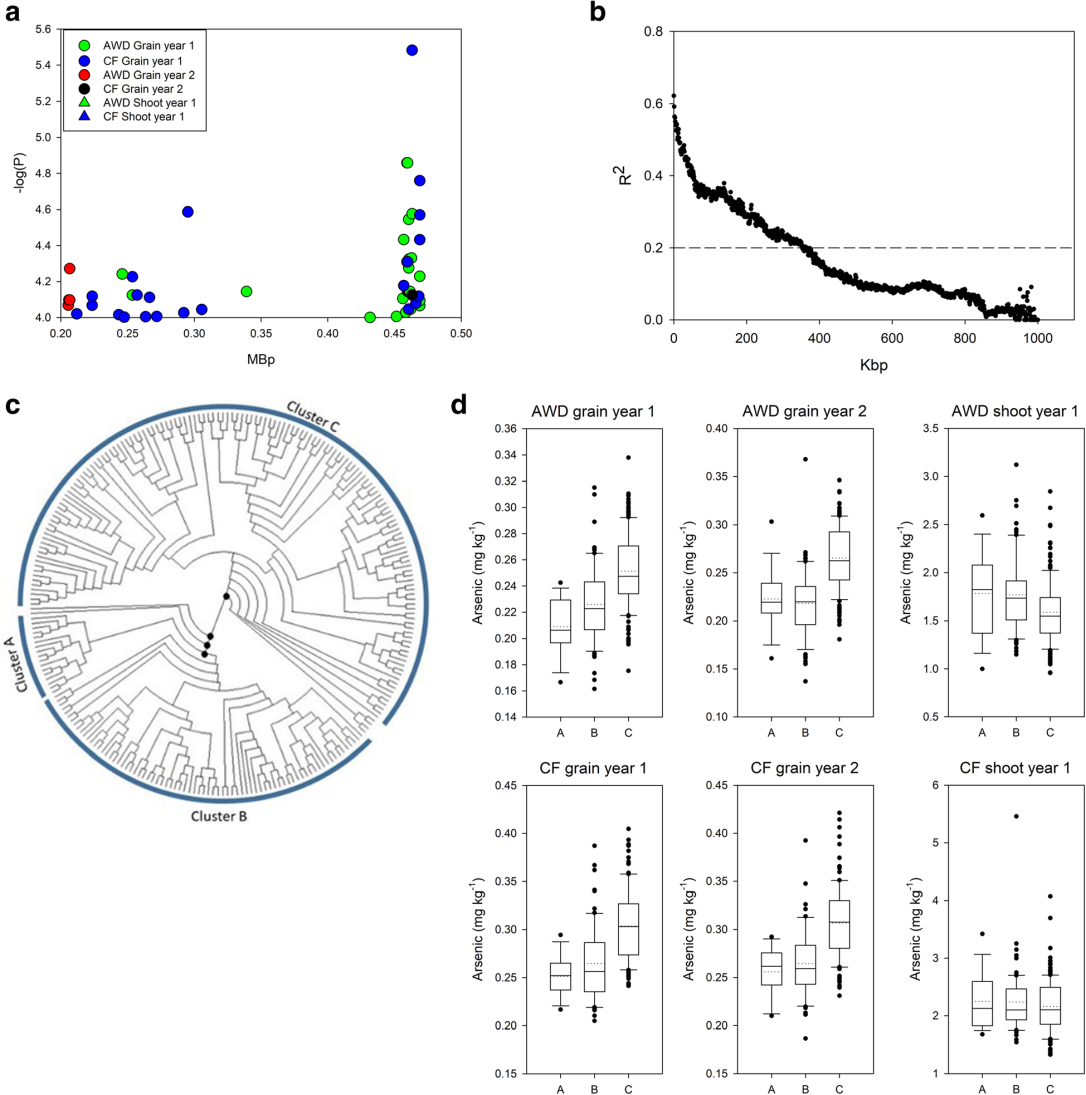
Significant associations for arsenic traits on chromosome 3 (0.20–0.47 Mb). **a** SNPs associated with the arsenic phenotypes. **b** Determination of local R^2^ on chromosome 3 from 0.0–1.0 Mb. **c** Neighbour-joining tree for the BAAP cultivars for all SNPs located between 0.20–0.47 Mbp on chromosome 3; black circles indicate node support of ≥50%. **d** Range of phenotypic variation observed for each cluster across each trait

On chromosome 3 between 0.21 and 0.47 Mbp a total of 68 SNPs were associated with grain arsenic under AWD in year 1 or year 2, or grain arsenic under CF in year 1. This is shown for clarity in Fig. 3a. No SNPs were associated with the shoot arsenic phenotypes (Additional file 3: Table S3, Fig. 3a). The calculated local LD decay from the top of chromosome 3 to 1 Mbp was determined to be 364 kbp (Fig. 3b). Based on this LD decay, all SNPs within the range of the QTL (0.21 to 0.47 Mbp) were used to identify genotype clusters for this region. Clustering analysis of the SNP data releveled 3 dominant clusters, with 17 accessions being assigned to cluster A, 75 accessions assigned to cluster B, and 150 accessions being assigned to cluster C (Fig. 3c, Additional file 3: Table S3); the remaining accessions could not be assigned to any of these clusters. For grain arsenic the C cluster plants had a higher average grain arsenic concentration (Fig. 3d), with these plants having a grain arsenic concentration between 16.6–17.7% higher compared to the plants from the clusters with the lowest grain arsenic (Additional file 3: Table S3). The plants that were from the A cluster had higher shoot arsenic compared to plants from the other clusters (Fig. 3d), being 4.0 and 10.9% higher than the lowest cluster (cluster C; Additional file 3: Table S3).

### Chromosome 5 ~ 0.32 Mbp

**Fig. 4:**
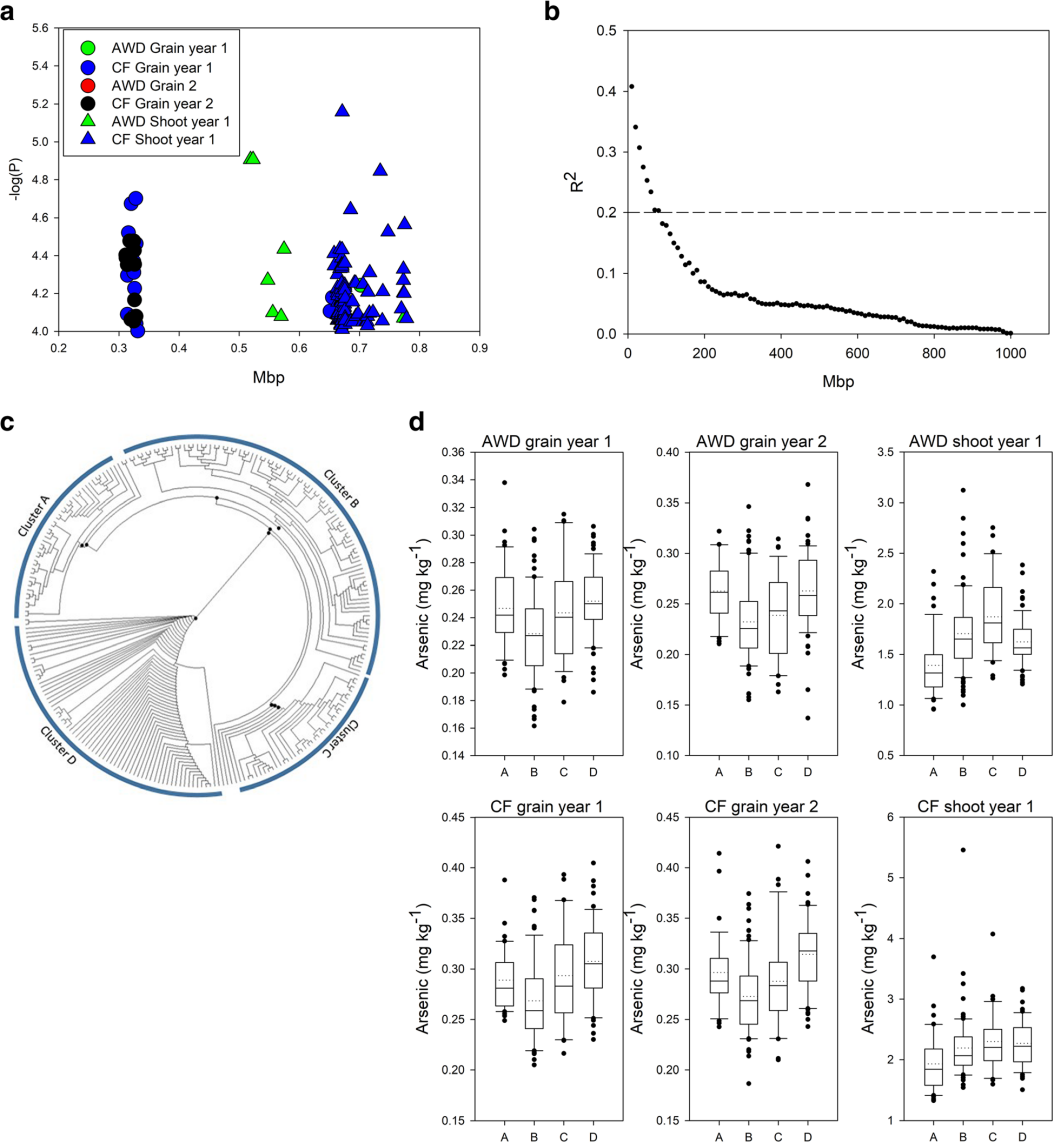
Significant associations for arsenic traits on chromosome 5 (0.20–0.90 Mb). **a** SNPs associated with the arsenic phenotypes. **b** Determination of local R^2^ on chromosome 5 from 0.0–1.0 Mb. **c** Neighbour-joining tree for the BAAP cultivars for all SNPs located between 0.31–0.33 Mbp on chromosome 5; black circles indicate node support of ≥50%. **d** Range of phenotypic variation observed for each cluster across each trait

On chromosome 5 between 0.31 and 0.33 Mbp a total of 31 SNPs were associated with grain arsenic under CF in year 1 or year 2 (Fig. 4a). No SNPs were associated with the grain arsenic under AWD phenotype or either of the shoot arsenic phenotypes in this region (Additional file 2: Table S2 and Additional file 3: Table S4, Fig. 4a). However, between 0.52–0.67 Mbp SNPs were associated with shoot arsenic under AWD and CF in year 1 (Fig. 4a and Table 1). In this region, from the top of chromosome 5 to 1 Mbp, the calculated local LD decay was determined to be approximately 80 kbp (Fig. 4b). This local LD decay suggests that SNPs between 0.52–0.67 represent a different locus to those between 0.31–0.33. All SNPS within the range of the CF grain QTL (0.31 to 0.33 Mbp) were used to determine genotype clusters. Clustering analysis of the SNP data revealed 4 clusters (Fig. 4c), with 44 accessions being assigned to cluster A, 87 accessions being assigned to cluster B, 39 accessions being assigned to cluster C, and 64 accessions being assigned to cluster D. The remaining accessions could not be assigned to any of these clusters. For grain arsenic the plants from cluster D had a higher average grain arsenic concentration compared to the plants from the other clusters (Fig. 4d), and in all cases the plants within cluster B had the lowest grain arsenic, with the plants within cluster D having between 9.5–13.1% higher grain arsenic concentration compared to the plants within cluster B (across years and treatments; Additional file 3: Table S4). The highest average shoot arsenic concentration was observed in the accessions within cluster C in both the CF and AWD treatment, while the plants within cluster A had the lowest average shoot arsenic (a reduction of 25.1 and 16.1% under AWD and CF respectively).

### Chromosome 4 ~ 31.7 Mbp

**Fig. 5:**
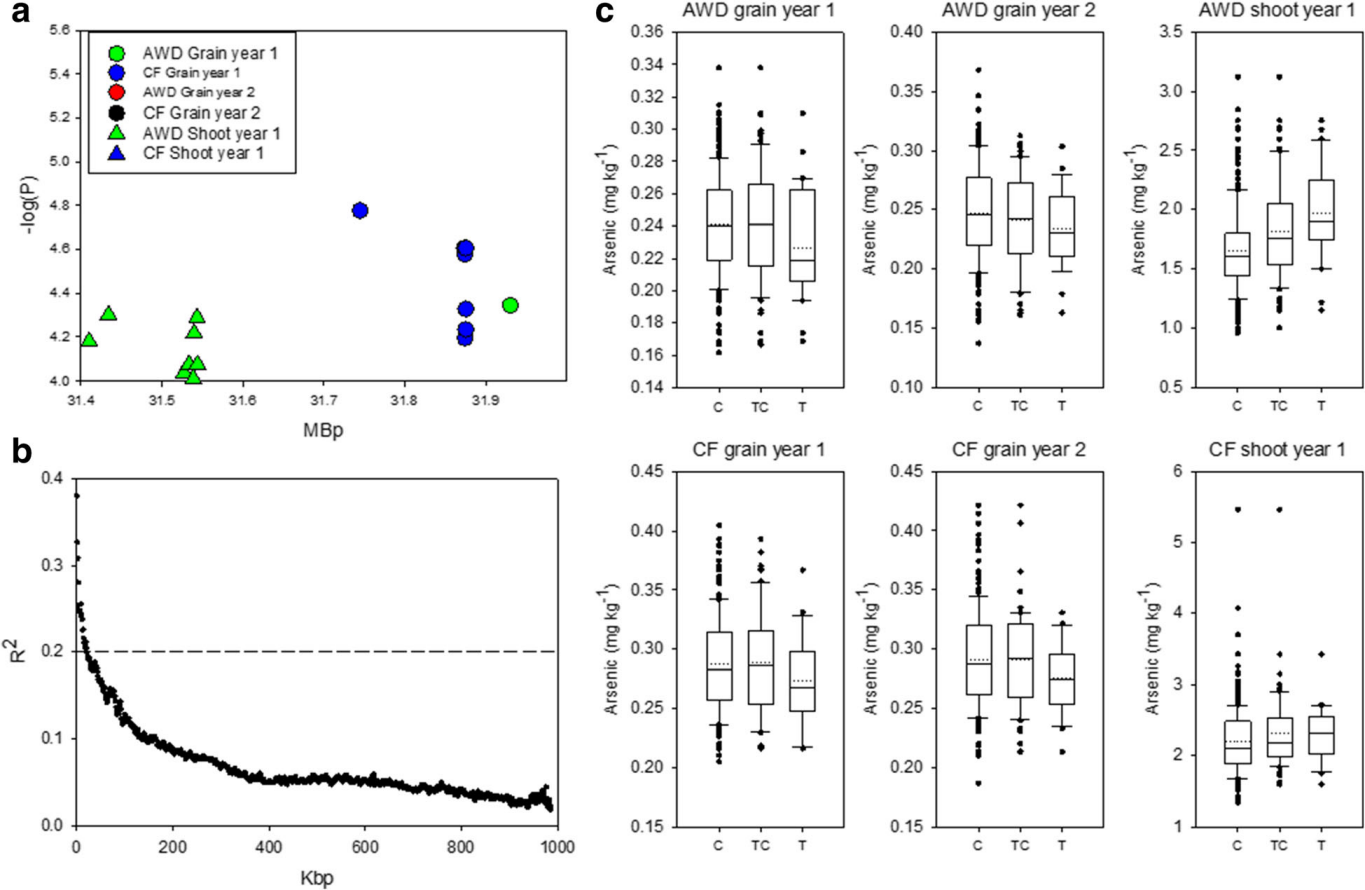
Significant associations for arsenic traits on chromosome 4 (31.4–32.0 Mb). **a** SNPs associated with the arsenic phenotypes. **b** Determination of local R^2^ on chromosome 4 from 31.0–32.0 Mb. **c** Range of phenotypic variation observed for each allele across each trait for SNP 4:31432484

On chromosome 4 between 31.41 and 31.87 Mbp a total of 26 SNPs were associated with either grain arsenic under AWD or CF in year 1, or shoot arsenic in the plants grown under AWD in year 1 (Fig. 5a). Analysis of local LD decay in this region identified that local LD decay was only ~ 20 kb (Fig. 5b), suggesting it is unlikely that this region (0.46 Mbp) is a single QTL, as the SNPs in this region spanned ~25x the local LD decay. As it is likely that the region contains more than one QTL based on the local LD decay determination, it did not seem logical to identify genotype clusters for the whole region. Therefore, instead of a cluster analysis, the effect size was determined for the most statistically significant associated SNP (across all experiments; SNP 4:31432484 at 314.43 Mbp) in the region. There were three alleles present at SNP 4:31432484, homozygous C (*n* = 172), homozygous T (*n* = 30), and heterozygous TC (*n* = 46). The variation between the alleles for the highest and lowest grain arsenic concentration ranged from 6.4–9.9%, and in four of the six experiments the highest observation for arsenic concentration was made in the plants with the heterozygous allele (Fig. 5c). However, the allele with the lowest arsenic concentration was variable across the different experiments (Additional file 3: Table S5).

### Assigning high yielding cultivars to genotype cluster form QTLs on chromosome 3 and 5

**Fig. 6:**
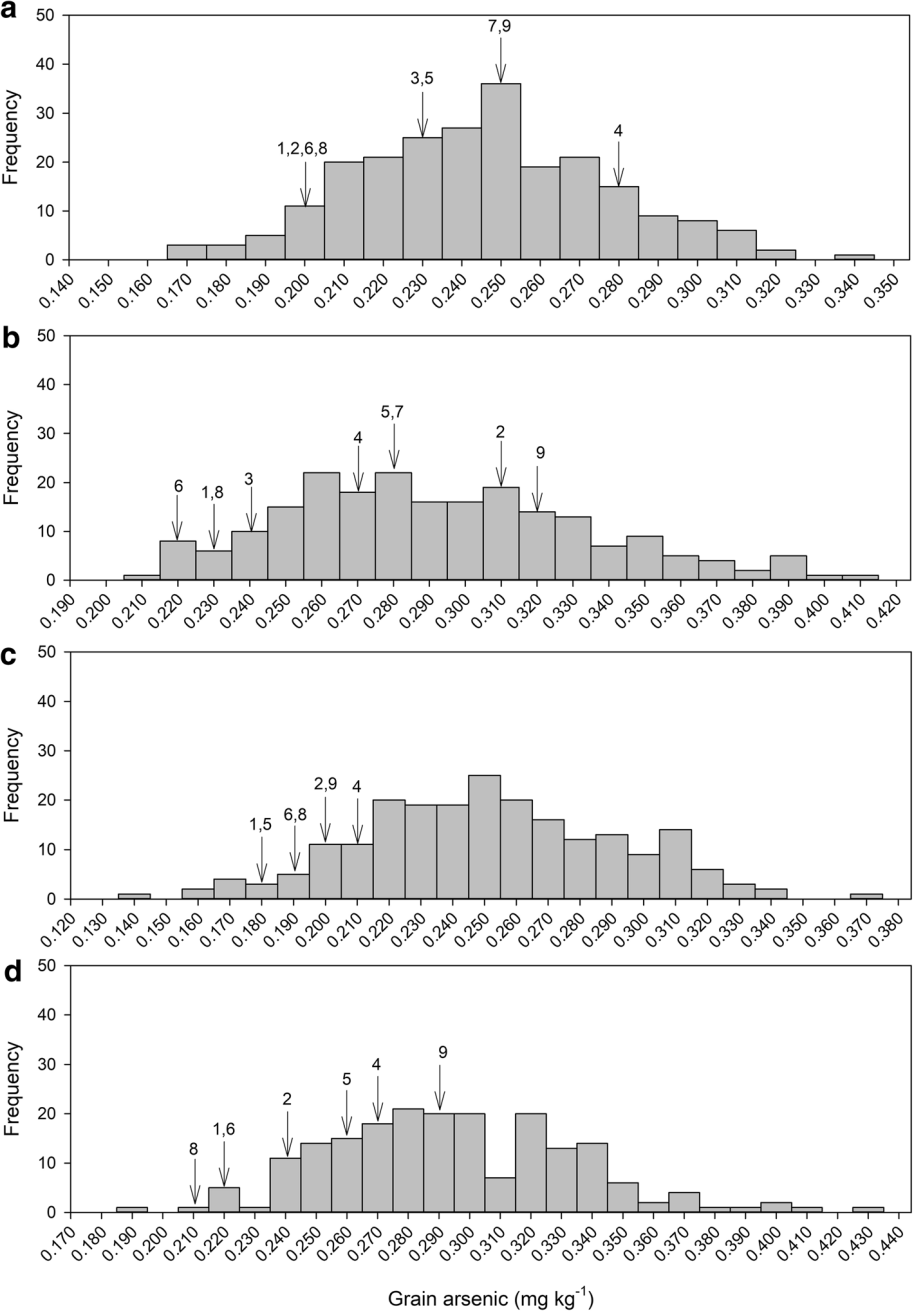
Grain arsenic concentration for the BAAP in the four experiments **a** AWD year 1. **b** CF year 1. **c** AWD year 2. **d** CF year 2. The grain arsenic concentration of the high yielding elite Bangladeshi cultivars is indicated by arrows 1) BR 3; 2) BR 6; 3) BR16; 4) BRRI dhan28; 5) BRRI dhan 45; 6) BRRI dhan 47; 7) BRRI dhan50; 8) BINA dhan5; 9) Iratom 24

In addition to the BAAP populations a number of high yielding Bangladeshi cultivars were tested for arsenic concentration in the field trial. These were BR 3, BR 6, BR 16, BINA dhan5, BRRI dhan28, BRRI dhan45, BRRI dhan47, BRRI dhan50, and Iratom 24. Generally these cultivars had below average grain arsenic concentration (Fig. 6), with BR 3 and BINA dhan5 having consistently low grain arsenic. However, Iratom 24 did have above average grain arsenic concentration in three out of the four experiments (Fig. 6). Using a number of informative SNPs (14 on chromosome 3 and 7 on chromosome 5) these cultivars were assigned to the genotype clusters identified for the QTLs on chromosome 3 and 5. This was not done for the chromosome 4 QTL because there was less confidence in the SNP used (in Fig. 5) being the informative of the functional allelic variation. For the QTL on chromosome 3: BR 6, BR 16, BINA dhan5, BRRI dhan28, BRRI dhan45, BRRI dhan47, and BRRI dhan50 all belonged to the cluster A, while BR3 and Iratom 24 were classified into cluster C. For the QTL on chromosome 5: BRRI dhan28, BR 16, and Iratom 24 belonged to cluster C, while BRRI dhan45, BRRI dhan47, BRRI dhan50, BR3, and BINA dhan5 belonged to cluster A. Based on the available sequence data BR 6 is not classifiable into any of the three clusters on chromosome 5.

## Discussion

In this study we have demonstrated that there are a large number of genomic loci that regulate the accumulation of arsenic in rice grains. Some of these loci are located next to known genes that have previously been shown to regulate arsenic accumulation, using combinations of transgenic and mutation approaches. By exploring the clustering of sequence information at these loci we have been able to explore the natural variation that regulates the accumulation of arsenic in rice grains.

### Impact of AWD

It has been widely reported that AWD alters the accumulation of a range of elements in rice grains including arsenic (Norton et al. 2017a; Chou et al. 2016; Linquist et al. 2015; Wang et al. 2014). The results of a small sub sample of this population (22 accessions of which 19 are in the BAAP) from this experiment and one further field study has been previously published (Norton et al. 2017b). The higher number of accessions reported here has increased the range of natural grain arsenic observed in the accessions under both AWD and CF (as seen in the range displayed in Table 2). Across the population mean grain arsenic was 18.7 and 15.2% greater in the accessions grown under CF. This decrease in arsenic for the accessions grown under AWD treatment reflect a majority of other studies (Chou et al. 2016; Linquist et al. 2015). Accessions SORIBHOG and Jhona 349 are notable cultivars as they were identified as being consistently low in grain arsenic across the experiments (Table 3). Of note is the weak or lack of genotype by management (treatment) interaction for grain arsenic concentration. In year 1 the interaction only explained 7.7% of the observed variation in grain arsenic concentration while in year 2 no interaction was detected (Table 2). This generally weak interaction is promising in terms of breeding for low grain arsenic, as it would indicate that accessions that have low grain arsenic in the CF treatment will also likely have low grain arsenic under AWD (Table 3). The weak/lack of genotype by management for grain arsenic concentration also indicates that the mechanisms for grain arsenic accumulation for the plants under AWD and CF are likely to be the same.

### Flowering time and grain arsenic

It has previously been demonstrated in genetic studies of grain arsenic accumulation in rice that flowering time has a strong impact on grain arsenic concentration (Norton et al. 2012, 2014). Here, in three of the four treatment and year combinations there was a relationship explaining up to 10.6% of the variation in grain arsenic, all following a similar trend where the later the flowering of the plant the lower the grain arsenic concentration (Additional file 4: Figure S1). The negative nature of the relationship between flowering time and grain arsenic matches that reported by Norton et al. (2012) for the Bala x Azucena mapping population grown in China, but the strong flowering time effect observed for grain arsenic on the Rice Diversity Panel 1 grown in multiple sites (Norton et al. 2014) was not generally linear as found here. It is presumed that the link is related to arsenic availability in soils and related uptake kinetics since grain inorganic arsenic is predominantly sourced from the soil rather than redistributed from other plant tissue (Carey et al. 2011). This agrees to some extent with the arsenic availability for the year 1 experiments which have been measured and reported (Norton et al. 2017a), where pore water arsenic concentration dropped markedly in the CF treatment between 20 and 30 days after transplanting, but not in the AWD treatment. Further investigation of the link between grain arsenic and flowering time is merited. Nevertheless, by deliberately limiting the degree of flowering time in the cultivar selection process when developing this population, the interference of phenology on the genetic dissection of grain arsenic QTLs is likely to be reduced compared to other populations. Examining the grain arsenic QTLs reported here for co-localisation with flowering time QTLs from the same experiment (reported in Norton et al. 2018) reveals overlap only for AWD year 2 at 1.38–2.14 on chromosome 3 (a QTL for flowering time in the same place for the same year and treatment), so the other arsenic QTLs reported here do not appear to be flowering time QTLs.

### QTLs for arsenic and the search for candidate genes

What is apparent from this study is the large number of loci detected (Additional file 2: Table S2). Like previous studies using other populations (e.g. Norton et al. 2014), many significant loci were detected for grain arsenic. What distinguishes this study is that here we have detected loci across multiple treatments and years. This has notable benefits when investigating these QTLs further; this treatment / year stability is essential when breeding rice with reduced arsenic as these accessions would be grown in a wide array of environments and under a diverse range of management approaches.

Whilst the global LD decay of 243 kbp (Norton et al. 2018) for this population was comparable with other rice GWA panels (Zhao et al. 2011; Kadam et al. 2017), this study clearly demonstrates that local LD decay is very variable across the genome. In the three regions that were investigated in detail the LD decay ranged between 20 kbp and 364 kbp. This large variability in the size of linkage blocks means that for each association a bespoke analysis pipeline is needed when identifying positional functional candidate genes. This could potentially limit high throughput analysis of traits which show a large number of significant association between the phenotype and the SNPs.

After GWA mapping there is a need to examine allelic variation in order to determine the effect of alleles on trait values and to help identify candidate genes. In some reports, trait values for the two classes of the most significant SNP are given. This probably does not allow full exploration of the likely allelic variation at the haplotype level as there is likely to be more than two alleles for the functional gene. Alternatively, where a strong candidate gene is located at the QTL, haplotype variation of that gene has been employed (e.g. Yang et al. 2018). This, however, is not possible if no candidate gene is obvious from examining genome annotation in the QTL region. Here, to determine the trait values of cultivars that are genetically similar at these QTLs, and therefore share similar haplotypes, a clustering approach was undertaken for three selected QTL regions. Local LD decay was used to define the genomic range to include in the clustering. This approach was adopted as a rapid way of assigning accessions to clusters and determining the scale of the effect on the genomic loci for the phenotype. For 2 of these loci (Figs. 3 and 4), this approach worked well and the effect size of haplotype groups was determined. However, for one loci this approach was less successful (QTL on chromosome 4, Fig. 5). For this loci, this was due to the very small estimated LD decay, as it appears that this QTL has a much larger span (in bp) than the calculated local LD decay. It must be noted that the LD decay within a region was determined using a 1 Mbp window, possibly hiding finer scale LD decay relationships. The approach of clustering accessions based on all the SNPs within a QTL region provides a better representation of the response of the cultivars that are genetically similar within a QTL region, rather than just using a single SNP with the QTL region (as this can only represent the difference for that single SNP).

### Candidate genes

Exploration of the QTL regions identified in this study has allowed for a number of candidate genes to be proposed for these QTLs. The gene Os03g01700 at 0.43 Mb is *Lsi2* and is within the physical region of the grain arsenic QTL (0.20–0.47 Mbp) detected in all experiments on chromosome 3 (Fig. 3). Lsi2 has been shown through mutant studies to regulate the flow of arsenite as well as silicon across the plasma membrane of roots and nodes, and to influence grain arsenic (Ma et al. 2008). In addition to this candidate gene on chromosome 3, there is a good positional candidate gene for the QTL that spans 31.4–31.9 Mbp on chromosome 4 (Fig. 5): Os04g52900, OsABCC1 (Song et al. 2014). Using knockouts in rice it has been shown that OsABCC1, a C-type ATP-binding cassette transporter, is involved in reducing arsenic concentration in rice grains (Song et al. 2014). It functions by sequestering arsenic in the vacuoles of the phloem companion cells of the nodes and therefore limiting arsenic into the grains (Song et al. 2014). OsABCC1 is located at 31.50–31.51 Mbp on chromosome 4, within the QTL position. However, at this genomic loci, the linkage equilibrium is very small (20 kb). The region that SNPs were detected in was much larger than the LD decay (25 times greater), which could indicate that the SNPs in this region could be associated with multiple QTLs, suggesting that linking this region of SNPs to the candidate gene OsABCC1 is complicated. Not all known transporters of arsenic were identified as co-localising with QTLs detected in this study. Thus, known transporters OsPT8 (Wang et al. 2016), OsNIP1;1, and OsNIP3;3 (Sun et al. 2018) are not located at QTLs detected here.

### Why has Lsi2 not been detected as a QTL before

In the BAAP there are 12 SNPs within *Lsi2*, all synonymous. Within clusters A and B identified here (Fig. 3) these 12 SNPs are invariable. However, 9 of the SNPs almost perfectly fit a differentiation with cluster C. Thus for SNP 3:430520 all accessions in clusters A and B are homozygous T while all but two in cluster C are homozygous G, the other two being heterozygotes. For SNP 3:430598 all accessions in clusters A and B are homozygous G while all but two in cluster C are homozygous A, with one being homozygous G and one being heterozygous. For SNP 3:432207 all accessions in clusters A and B are homozygous A and all in cluster C are homozygous G. This provides supporting evidence that polymorphism within *Lsi2* is associated with grain and shoot arsenic and is a good candidate for the causal gene. However, it would be reasonable to question why this association has not been detected before in biallelic mapping studies or GWAS. It seems likely that the biallelic studies conducted on Bala x Azucena by the authors (Norton et al. 2010 and Norton et al. 2012) would not have revealed this since examining the sequences (Fastq data have been deposited in the NCBI Short Read Archive Acc_ID SRA050654.1) suggests both parental cultivars have the same allele, which is the one shared with the reference genome of Nipponbare. Considering the study on the Rice Diversity Panel (Norton et al. 2014), it appears that polymorphism in *Lsi2* matches the *Indica*/*Japonica* divide in rice, is almost invariable in *Japonica*, and is most balanced in the *aus* subpopulation. In the SNP-Seek database for the High Density Array, only six SNPs are identified within *Lsi2*; none are non-synonymous and only one of them is also identified in the BAAP, at 3:432207. This is one of the SNPs in perfect agreement with the clustering revealed in this genomic region that is associated with differing arsenic concentration (Fig. 3). In RDP1 for this SNP *aus* are 39 homozygous A, and 14 homozygous G (39/14), while for *aromatic* it is 10/0 (with one heterozygous), indica 12/57, *temperate japonica* 90/0 (two heterozygous), and *tropical japonica* 80/1 (three heterozygous). This information strongly suggests the functional polymorphism that is associated with different arsenic concentration is also associated with the *Indica*/*Japonica* split within rice, with the *Japonica* allele lowering grain arsenic. It also suggests *aus* is the most balanced of the genetic subgroups, making it the most suitable to detect the polymorphism, with minor allele frequency of 26% compared to 17% for *indica* and an absence of the *indica* allele in the other subgroups. The best chances to detect it would be in a large *aus* population like the BAAP. This suggests the BAAP is a superior population for detecting association between polymorphism in *Lsi2* and grain arsenic.

### Implications for breeding

While the identification of genotype clusters which have low grain arsenic is promising for breeding it is important to understand what alleles are present in the current elite high yield cultivars. In this study nine commonly cultivated Bangladeshi cultivars were grown in addition to the BAAP, and these were then assigned to the genotype clusters for the QTLs on chromosome 3 and 5. For the QTL on chromosome 3, a majority of the Bangladeshi varieties had the cluster that was identified as the low arsenic cluster, which would suggest that there is no scope for reducing grain arsenic in these cultivars using this QTL (in other words they appear to have the *japonica* allele for *Lsi2*). However, two cultivars (BR 3 and Iratom 24) were identified as belonging to the C cluster, and for these cultivars (based on the average of the BAAP) incorporation of the cluster A allele at this location could reduce grain arsenic by ~ 16%. On chromosome 5 none of the cultivars belong to the low grain arsenic cluster, so this would suggest that if this low grain arsenic cluster could be incorporated into these high yielding cultivars, grain arsenic concentration could be lowered by ~ 10%.

In the analysis above, only a small number of apparently stable QTLs are considered, while the study reveals 74 putative QTLs detected in at least one treatment and year. The implication is that there are multiple small effect loci that contribute to grain arsenic that are either difficult to detect or environmentally variable. These would not be easy to use in breeding using conventional marker assisted selection approach but genomic selection might render them tractable. In genomic selection, models predict breeding value based on marker-trait associations that encompasses all impacting loci, and requires a training population to derive the equation that is then applied to the breeding material (eg Lin et al. 2014). Genomic selection has not been routinely applied to crop breeding for ionomic traits but has great promise if the environmental stability of the small QTLs does not undermine it. The application of genomic selection for grain arsenic should be explored as an alternative route to exploit the results presented here.

## Conclusion

This study further supports the evidence that implementation of AWD will reduce the concentration of arsenic in rice grains. While confirmation of the proposed candidate genes is important, and provides confidence in the approach taken, a major research direction from this study will be in exploration of those QTLs that are identified consistently across years and treatments, and for which no positional candidate genes can readily be identified. While genes that regulate arsenic in rice plants are rapidly being identified (e.g. Ma et al. 2008; Song et al. 2014; Sun et al. 2018) using mutant screens and transgenic approaches, the identification of novel genes based on natural variation (which is extensive in rice for these traits) has not advanced as quickly. Importantly, the observation that the introduction of the arsenic QTL on chromosome 5 will reduce grain arsenic in the elite high yielding cultivars currently grown in Bangladesh indicates how marker assisted breeding can be used to reduce arsenic in rice, therefore reducing arsenic entering the diet.

## Methods

### Population and field screen

The population used in this study was the Bengal and Assam Aus Panel (BAAP) (Norton et al. 2018). The population consists of 266 landraces identified as belonging to the *aus* subpopulation, as well as a number of cultivars from the OryzaSNP panel (McNally et al. 2009) and a number of high yielding elite Bangladeshi cultivars (Norton et al. 2018); these were BR 3, BR 6, BR 16, BINA dhan5, BRRI dhan28, BRRI dhan45, BRRI dhan47, BRRI dhan50 and Iratom 24. The 266 *aus* cultivars from the population have been genotyped, and a 2 million SNP database constructed (Norton et al. 2018). Sequence data is also available for the high yielding elite Bangladeshi cultivars.

The BAAP was grown in the field in Mymensingh, Bangladesh in the Boro (dry season) of 2013 (referred to as year 1) and 2014 (referred to as year 2). The population was screened under both AWD and continuously flooded (CF) conditions. The full details of the field screening is given in Norton et al. (2017a) and Norton et al. (2018), a summary of soil properties is given in Additional file 3: Table S6. In both years the seeds were initially grown in a nursery bed and transplanted to the field plot after 44 days in 2013 and 51 days in 2014. Flowering time is calculated from time of sowing. The plants were transplanted into 8 experimental plots with two plants per hill, a distance of 20 cm between each hill in a row, and a 20 cm distance between each row of 4 m length. Rice accessions were planted in single rows, with a check cultivar BRRI Dhan 28 transplanted into each alternate row. After transplanting, the plots were flooded. For the four CF plots the surface water was kept at a depth of between 2 cm and 5 cm above the soil surface during the vegetative and reproductive stage (13th April 2013). For the four AWD plots plastic perforated tubes (pani pipe) were placed across the plots to monitor the water depth. The aim was to allow water to drain / percolate naturally from the AWD plots until the average depth of the water was 15 cm below the soil surface. At that point the plots were irrigated to bring the water depth to between 2 cm and 5 cm above the soil surface (Norton et al. 2017a). AWD was applied only from 14 days after transplanting until flowering. Once the cultivars had flowered and the grains matured, the grain and shoots from every cultivar was hand harvested from the six central hills of each row.

### Arsenic analysis

Shoot and grain arsenic were determined as described in Norton et al., 2017a. Briefly, rice grains were de-husked and oven dried, followed by microwave digestion with concentrated nitric acid and hydrogen peroxide as described in Norton et al. (2012). Straw was oven dried, powdered, and digested using nitric acid and hydrogen peroxide on a block digester. Total arsenic analysis was performed by inductively coupled plasma – mass spectroscopy (ICP-MS). Trace element grade reagents were used for all digests, and for quality control replicates of certified reference material (CRM) (Oriental basma tobacco leaves [INCT-OBTL-5]) and rice flour [NIST 1568b]) were used; blanks were also included. All samples and standards contained 10 μg L^− 1^ indium as the internal standard.

### Genome-wide association (GWA) mapping

GWA mapping was conducted using “PIQUE” (Parallel Identification of QTL’s Using EMMAX, https://github.com/tony-travis/PIQUE) to pre-process genotype and phenotype data and then run association analysis for each phenotype in parallel using EMMAX (Kang et al. 2010) as described in Norton et al. (2018).

### Clump analysis of significant SNPs

Significant SNPs were binned together into peaks using a sliding window based on the decay of linkage disequilibrium (LD) using the PLINK (Purcell et al. 2007) with parameters “--clumpp1 0.0001 --clump-p2 0.0001 --clump-r2 0.3 --clump-kb 243”. For every SNP with *P* < 0.0001, pairwise r^2^-values were calculated between surrounding SNPs that (1) fell within 243 kb and (2) had a *P* < 0.0001; any two SNPs meeting this criteria that also shared an r^2^ ≥ 0.3 were clumped into bins. Binning was done for each experiment for each trait measured. Singleton significant SNPs (*P* < 0.0001) were discarded if no other SNP within the LD window had a *P* < 0.0001. The LD decay value of 243 kb was selected as this is the average LD decay for the population (Norton et al. 2018).

### Determination of local linkage disequilibrium

Local LD decay was estimated as r^2^ (VanLiere and Rosenberg 2008) using PLINK (Purcell et al. 2007). Local LD decay within a 1 Mb genomic region was determined using the most statistically significant SNP as the central SNP within the window. Pairwise r^2^ values between all SNPs was calculated and LD decay values were binned (10 kb bins) based on the distance between the SNPs.

### Assignment of genotype clusters within candidate regions

To determine the response of cultivars that share similar genomic regions with an association a neighbourhood joining tree (NJT) approach was taken to identify cultivars that share a similar genetic background within the QTL. Once identified the cultivars were then assigned to a cluster (specific to that QTL region) and then the phenotypic response for the cultivars within each cluster was observed. NJT were bootstrapped 100 times from a distance matrix using TreeBest (Ruan et al. 2008) and clusters of genotypes were identified at a bootstrap support value of 50% or greater. Clusters with less than 5 accessions are not reported. The SNPs used to generate the NJT were all the SNPs identified in the QTL range identified using CLUMP.

### Assigning high yielding cultivars to cluster groups

For the cluster on chromosome 3, 14 representative SNPs were identified and selected, representing the three identified clusters (SNP 203791, 204280, 211702, 212013, 269583, 269595, 296784, 305558, 345938, 346122, 353817, 400758, 431326, and 432207). SNPs were then called at the SNP locations for the nine high yielding elite Bangladeshi cultivars, based on the SNPs being assigned to the identified clusters. This process was also done for the QTL on chromosome 5 using seven representative SNPs (311397, 314001, 320483, 315688, 321371, 324848, and 325772).

### Statistical analysis

All statistical analyses were performed using the statistical software Minitab v.17 (State College PA) and SigmaPlot v.13 (Systat Software Inc., CA) and significance reported at alpha < 0.05. Assumptions of normality were tested using the Anderson-Darling test in Minitab.

## Additional files

Additional file 1: **Table S1.** Phenotype data used for genome wide association analysis. (XLSX 39 kb)

Additional file 2: **Table S2.** Clump analysis of the significant SNPs associated with grain and shoot arsenic concentration. (XLSX 19 kb)

Additional file 3: **Table S3.** Mean arsenic concentrations for the different traits across the 3 different genotype clusters identified on chromosome 3 between 0.20–0.47 Mb. Values in bold and underlined are the highest and lowest mean value for that trait across the clusters, respectively. **Table S4.** Mean arsenic concentrations for the different traits across the 4 different clusters of genotypes identified on chromosome 5 between 0.31–0.33 Mb. Values in bold and underlined are the highest and lowest mean value for that trait for that cluster of genotypes. **Table S5.** Mean arsenic concentrations for the different traits across the 3 different alleles for SNP 4:31432484 genotypes on chromosome 4 at 31.4 Mbp. Values in bold and underlined are the highest and lowest mean value for that trait for that allele. **Table S6.** Physical and chemical properties of the soil. Adapted from Norton et al. (2017a) and Hossain et al. (2009). (DOCX 19 kb)

Additional file 4: **Figure S1.** Relationship between flowering time and grain arsenic concentration for the four experiments; a) AWD year 1, b) CF year 1, c) AWD year 2, d) CF year 2. (JPG 1990 kb)

## Data Availability

The 2 M SNP genotype data of the BAAP is available as a project called “BAAP” at the SNP-Seek database (http://snp-seek.irri.org/). The phenotype data used for the GWAS is presented in Additional file 1: Table S1.
