# Supplementary material for: Genetic loci regulating arsenic content in rice grains when grown flooded or under alternative wetting and drying irrigation
Source: Rice (N Y). 2019 Jul 22;12:54. doi: 10.1186/s12284-019-0307-9 (PMC6646650; doi:10.1186/s12284-019-0307-9)
Supplement: Supplementary file 3 — Table S3. Mean arsenic concentrations for the different traits across the 3 different genotype clusters identified on chromosome 3 between 0.20–0.47 Mb. Values in bold and underlined are the highest and lowest mean value for that trait across the clusters, respectively. Table S4. Mean arsenic concentrations for the different traits across the 4 different clusters of genotypes identified on chromosome 5 between 0.31–0.33 Mb. Values in bold and underlined are the highest and lowest mean value for that trait for that cluster of genotypes. Table S5. Mean arsenic concentrations for the different traits across the 3 different alleles for SNP 4:31432484 genotypes on chromosome 4 at 31.4 Mbp. Values in bold and underlined are the highest and lowest mean value for that trait for that allele. Table S6. Physical and chemical properties of the soil. Adapted from Norton et al. (2017a) and Hossain et al. (2009). (DOCX 19 kb) [file 12284_2019_307_MOESM3_ESM.docx]

Additional file 3: Table S3. Mean arsenic concentrations for the different traits across the 3 different genotype clusters identified on chromosome 3 between 0.20-0.47 Mb Mb. Values in bold and underlined are the highest and lowest mean value for that trait across the clusters, respectively.

|  | Arsenic concentration (mg kg^-1^) | | | | | | |
| --- | --- | --- | --- | --- | --- | --- | --- |
|  | AWD | | |  | CF | | |
| Cluster (number of genotypes within that cluster | Grain year 1 | Grain year 2 | Shoot |  | Grain year 1 | Grain year 2 | Shoot |
| A (17) | 0.209 | 0.223 | **1.782** |  | 0.251 | 0.256 | **2.25** |
| B (75) | 0.226 | 0.218 | 1.769 |  | 0.265 | 0.264 | 2.237 |
| C (150) | **0.251** | **0.265** | 1.587 |  | **0.304** | **0.307** | 2.160 |
| % difference between clusters* | 16.7 | 17.7 | 10.9 |  | 17.4 | 16.6 | 4.0 |

*between the lowest and highest clusters

Additional file 3: Table S4. Mean arsenic concentrations for the different traits across the 4 different clusters of genotypes identified on chromosome 5 between 0.31-0.33 Mb. Values in bold and underlined are the highest and lowest mean value for that trait for that cluster of genotypes.

|  | Arsenic concentration (mg kg^-1^) | | | | | | |
| --- | --- | --- | --- | --- | --- | --- | --- |
|  | AWD | | |  | CF | | |
| Cluster (number of genotypes within that cluster | Grain year 1 | Grain year 2 | Shoot |  | Grain year 1 | Grain year 2 | Shoot |
| A (44) | 0.247 | **0.263** | 1.393 |  | 0.289 | 0.296 | 1.930 |
| B (87) | 0.228 | 0.232 | 1.704 |  | 0.268 | 0.273 | 2.193 |
| C (39) | 0.244 | 0.239 | **1.871** |  | 0.293 | 0.288 | **2.300** |
| D (64) | **0.252** | **0.263** | 1.622 |  | **0.308** | **0.314** | 2.270 |
| % difference between clusters* | 9.5 | 11.8 | 25.5 |  | 13.0 | 13.1 | 16.1 |

*between the lowest and highest clusters

Additional file 3: Table S5. Mean arsenic concentrations for the different traits across the 3 different alleles for SNP 4:31432484 genotypes on chromosome 4 at 31.4 Mbp. Values in bold and underlined are the highest and lowest mean value for that trait for that allele.

|  | Arsenic concentration (mg kg^-1^) | | | | | | |
| --- | --- | --- | --- | --- | --- | --- | --- |
|  | AWD | | |  | CF | | |
| Cluster (number of genotypes within that cluster | Grain year 1 | Grain year 2 | Shoot |  | Grain year 1 | Grain year 2 | Shoot |
| C (172) | 0.241 | **0.250** | 1.584 |  | 0.287 | 0.292 | 2.139 |
| TC (46) | **0.251** | 0.234 | 1.718 |  | **0.298** | **0.298** | **2.307** |
| T (30) | 0.226 | 0.246 | **1.965** |  | 0.273 | 0.276 | 2.306 |
| % difference between alleles* | 9.9 | 6.4 | 19.4 |  | 8.4 | 7.4 | 7.3 |

*between the lowest and highest allele

Additional file 3: Table S6. Physical and chemical properties of the soil. Adapted from Norton et al., (2017a) and Hossain et al. (2009).

| Soil properties |  |
| --- | --- |
| Texture |  |
| Sand size particles (%)^*^ | 10.4 |
| Silt size particles (%)^*^ | 29.2 |
| Clay size particles (%)^*^ | 60.4 |
|  |  |
| pH^*^ | 6.6 |
|  |  |
| Organic C (%)^#^ | 0.93 |
| Total N (%)^#^ | 0.1 |
|  |  |
| Available S (mg kg^−1^)^#^ | 13 |
| Total Fe oxide (mg kg^−1^)^#^ | 7300 |
| Total P (mg kg^−1^)^*^ | 574 |
| Total Cr (mg kg^−1^)^*^ | 574 |
| Total Mn (mg kg^−1^)^*^ | 665 |
| Total Co (mg kg^−1^)^*^ | 665 |
| Total Ni (mg kg^−1^)^*^ | 51.2 |
| Total Cu (mg kg^−1^)^*^ | 40.7 |
| Total Zn (mg kg^−1^)^*^ | 99.7 |
| Total As (mg kg^−1^)^*^ | 4.63 |
| Total Mo (mg kg^−1^)^*^ | 0.57 |
| Total Cd (mg kg^−1^)^*^ | 0.19 |

* Norton et al (2017a); ^#^ Hossain et al. (2009).
