# Supplementary figures and images for: Genetic loci regulating arsenic content in rice grains when grown flooded or under alternative wetting and drying irrigation
Source: Rice (N Y). 2019 Jul 22;12:54. doi: 10.1186/s12284-019-0307-9 (PMC6646650; doi:10.1186/s12284-019-0307-9)

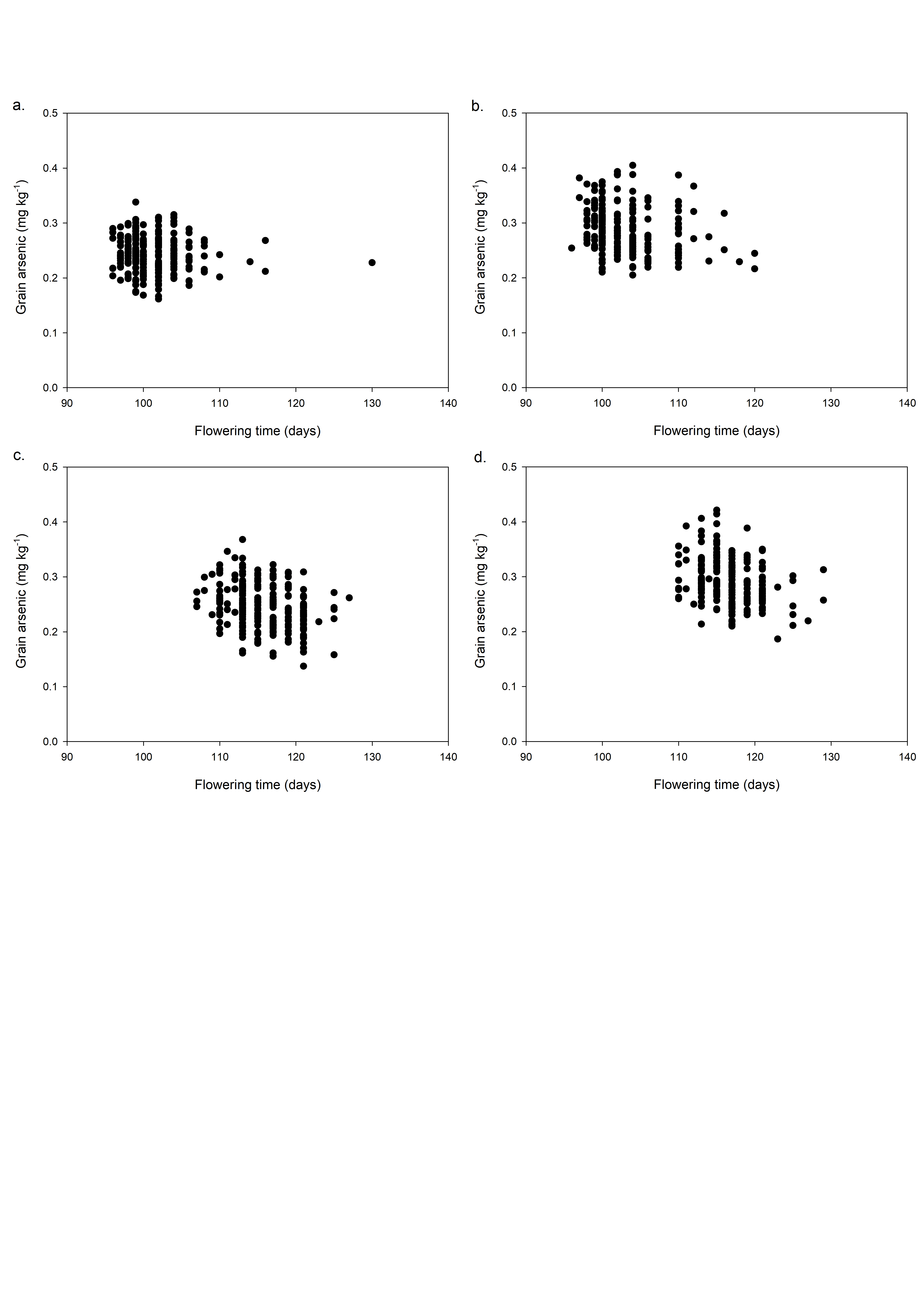

Supplement: Supplementary file 4 — Figure S1. Relationship between flowering time and grain arsenic concentration for the four experiments; a) AWD year 1, b) CF year 1, c) AWD year 2, d) CF year 2. (JPG 1990 kb) [file 12284_2019_307_MOESM4_ESM.jpg]
